# Sero-Survey of Polio Antibodies during Wild Poliovirus Outbreak in Southern Xinjiang Uygur Autonomous Region, China

**DOI:** 10.1371/journal.pone.0080069

**Published:** 2014-07-03

**Authors:** Hai-Bo Wang, Shuang-Li Zhu, Jing-Shan Zheng, Ai-Li Gou, Hui Cui, Yong Zhang, Gui-Jun Ning, Chun-Xiang Fan, Yuan-Sheng Chen, Ke-Li Li, Ping Yuan, Chao Ma, Jing Ma, Hui Zheng, Xin-Chun Fan, Xin-Lan Li, Hai-Shu Tang, Xiao-Lei Li, Fan Zhang, Dong-Mei Yan, Dong-Yan Wang, Zhi-Qiang Cui, Li-Ping Ren, Hui Zhu, Hui-Ling Wang, Xiao-Hong Jiang, Hong-Qiu An, Yang Liu, Jing Li, Wen-Bo Xu, Ning Wen, Ai-Qiang Xu, Hui-Ming Luo

**Affiliations:** 1 Expanded Program on Immunization, Chinese Center for Disease Control and Prevention, Beijing, China; 2 National Institute for Viral Disease Control and Prevention, Chinese Center for Disease Control and Prevention, Beijing, China; 3 Expanded Program on Immunization, Xinjiang Uygur autonomous region Center for Disease Control and Prevention, Urumqi city, Xinjiang Uygur autonomous region, China; 4 Hunan Provincial Center for Disease Control and Prevention, Changsha, China; 5 Hebei Provincial Center for Disease Control and Prevention, Shijiazhuang, China; 6 Liaoning Provincial Center for Disease Control and Prevention, Shenyang, China; 7 Tianjin Center for Disease Control and Prevention, Tianjin, China; 8 Shandong University Institute for Prevention Medicine, Shandong Provincial Key Laboratory of Infectious Diseases Control and Prevention, Shandong Center for Disease Control and Prevention, Jinan city, Shandong Province, China; Centers for Disease Control and Prevention, United States of America

## Abstract

**Background:**

After being polio free for more than 10 years, an outbreak following importation of wild poliovirus (WPV) was confirmed in Xinjiang Uygur Autonomous Region, China, in 2011.

**Methods:**

A cross-sectional study was conducted prior to supplementary immunization activities (SIAs), immediately after the confirmation of the WPV outbreak. In selected prefectures, participants aged ≤60 years old who visited hospitals at county-level or above to have their blood drawn for reasons not related to the study, were invited to participate in our study. Antibody titers ≥8 were considered positive.

**Results:**

Among the 2,611 participants enrolled, 2,253 (86.3%), 2,283 (87.4%), and 1,989 (76.2%) were seropositive to P1, P2 and P3 respectively, and 1744 (66.8%) participants were seropositive to all the three serotypes. Lower antibody seropositivities and geometric mean titers were observed in children <1 year of age and in adults aged 15–39 years.

**Conclusion:**

Serosurveys to estimate population immunity in districts at high risk of polio importation might be useful to gauge underlying population immunity gaps to polio and possibly to guide preparedness and response planning. Consideration should be given to older children and adults during polio risk assessment planning and outbreak response.

## Introduction

In 1988, the World Health Assembly resolved to eradicate poliomyelitis worldwide [1], [2]. Subsequently, the reported number of wild polioviruses (WPVs) cases was reduced from an estimated 350,000 in 1988 to 650 reported cases in 2011, and transmission of type 2 WPV was last observed in October 1999 [3], [4]. Despite such significant progress, circulation of indigenous WPV continues in three countries (Afghanistan, Nigeria, and Pakistan) in 2012, and WPV importation from remaining polio-endemic countries into polio-free areas has had a great challenge on global WPV eradication [2], [5]–[9]. In 2011, 11 WPV outbreaks occurred globally, including nine new outbreaks in eight countries and two outbreaks representing transmission from 2010 that continued into 2011 [4].

Poliomyelitis had been historically endemic and widely spread in China since the early 1950s, with about 20,000 paralytic cases reported annually since being incorporated into the national disease surveillance system in 1953. Polio eradication had been an important public health priority for the newly founded People's Republic of China. Under governnment leadership, a great many of efforts had been carried out to control polio, such as developing native oral attenuated poliovirus vaccine (OPV) and including OPV into Expanded Programme on Immunizations in 1978, constructing cold chain systems and strengthening regular immunization services, the number of poliomyelitis cases has declined dramatically. The implementation of Supplementary Immunization Activities (SIAs) since 1990, has resulted in significant progression of polio eradication. SIAs are a useful way to improve the herd immunity in a short term by providing immunization to the target population on a planned schedule, especially for these persons who are had to reach by routine immunization, and SIAs are also a supplement to routine immunization programs. SIAs are usually conducted in areas with poor routine immunization for eradicating or controlling vaccine preventable disease. The last indigenous WPV in China was isolated in September 1994 and China was certified as polio-free in October 2000 [10]–[12].

China, which shares borders with 2 of the remaining 3 countries that as of 2012 had never interrupted WPV transmission, has experience three instances of WPV importations between 1995 and 1999: 1995 and 1996 in Yunnan Province [13], and 1999 in Qinghai Province [10], [14], [15]. WPV importation and subsequent transmission will continue will continue to occur until endemic WPV transmission is interrupted globally.

After being polio free for more than 10 years, on Aug 25, 2011, an outbreak following importation of WPV originated from neighboring Pakistan was confirmed in Xinjiang Uygur Autonomous Region, China [4], [16]. Twenty-one WPV cases and 23 clinically compatible polio cases were identified in southern Xinjiang (Hotan, Kashgar, Bayinguole and Akesu) [16]. To assess overall population immunity and guide establishment of preparedness and response plan, a serological study was designed before SIAs to determine the prevalence of antibodies against poliovirus serotype 1 (P1), 2 (P2) and 3 (P3) in southern Xinjiang Uygur Autonomous Region, where the WPV epidemic was limited.

## Materials and Methods

### Study Participants

In 2011, immediately after the confirmation of WPV importation (25^th^ August), we conducted a serological survey in southern Xinjiang Uygur Autonomous Region between 27^th^ August and 6^th^ September before SIAs were conducted (8^th^ September). In southern prefectures, all residents aged ≤60 years old were eligible for inclusion. Participants who visited hospitals at the county-level or above for a blood extraction for reasons not related to polio investigation were invited to take part. Willing participants were enrolled in the study only after written, informed consent was provided by all participants of legal age (≥18 years) and by the parents or legal guardian for participants under 18 years of age. Individuals were excluded if they had a known immunodeficiency or had been treated with immunosuppressant drugs during the previous 12 months. Before SIAs were conducted, in the counties where WPV was isolated from cases/contacts/healthy persons or clinically compatible polio case was diagnosed, at least 50 serum samples were collected. This study was reviewed and approved by the Chinese Center for Disease Control and Prevention institutional review board.

### Measurement of antibody levels

Approximately 2-ml blood sample was collected for the titration of anti-poliovirus antibodies from each subject by venipuncture. Samples were immediately placed in an ice box and transported to the laboratory where serum was prepared within 24 h of drawing, and then stored at −20°C. Neutralization antibody titres against P1, P2, and P3 were measured by a microneutralization assay in accordance with the WHO standard procedure [17]. The maximum dilution of serum that protected at least 50% of test cells from viral lysis was determined. A serum sample with a titre of ≥1∶8 was considered positive and indicative of immunity.

### Statistical Analysis

Chi-square tests were used to evaluate the association between antibody seropositivity, age, sex and district where the participants lived. Wilcoxon test was used to compare the age difference among different prefectures. Analysis of variance (ANOVA) was used to compare the difference of geometric mean titres (GMTs) after logarithm transformation among different groups. If a statistical difference was present, Student-Newman-Kelus (SNK) post-hoc tests were performed to detect the inter-group difference. Statistical tests were performed using SAS 9.1 software (SAS Institute Inc, Cary, NC). The level of statistically significance chosen for all analyses was *p*<0.05.

## Results

### Outbreak profiles

**Figure 1:**
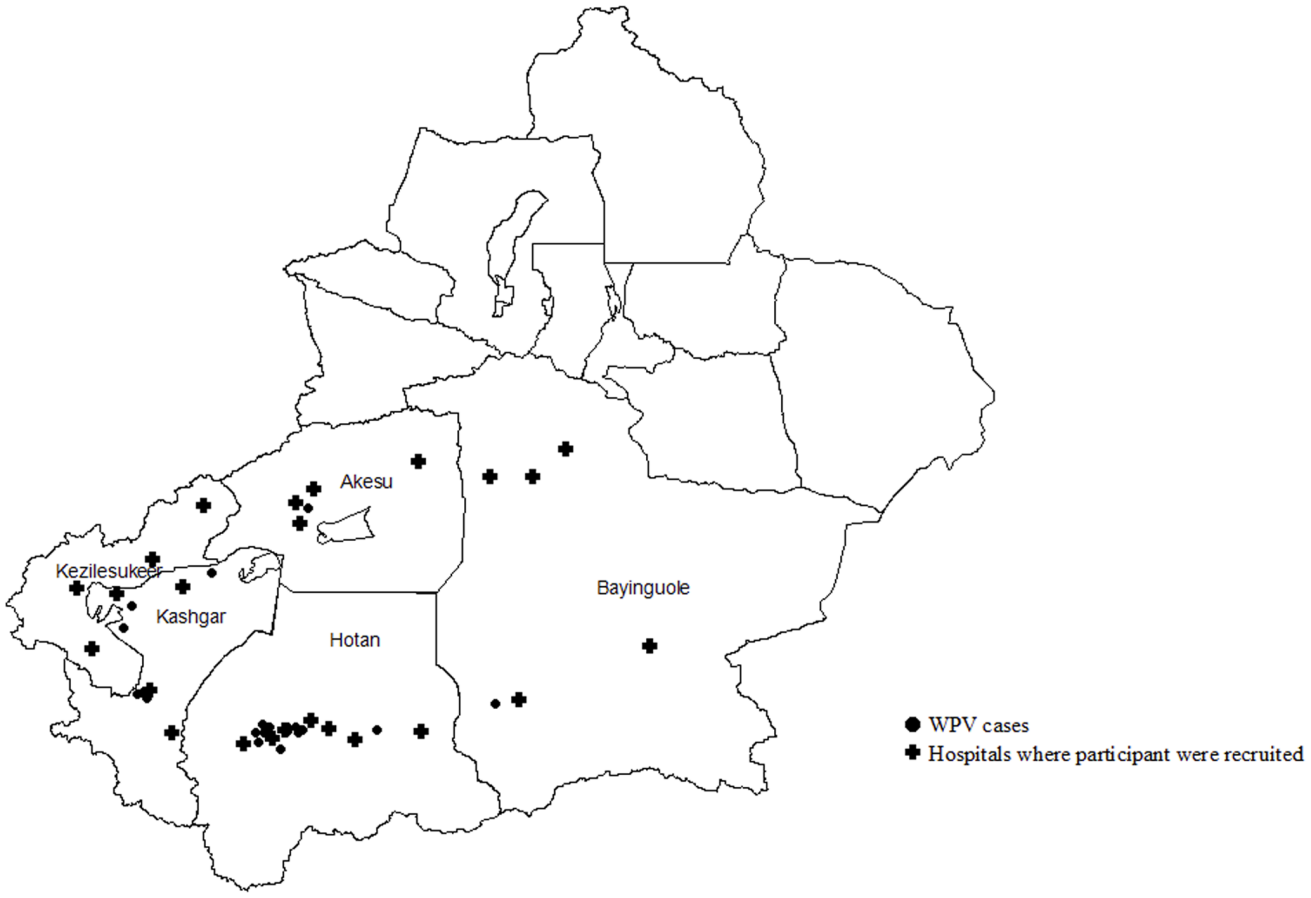
Distribution of WPV cases and hospitals in which blood specimens were collected in southern Xinjiang Uygur Autonomous Region.

Between July 3 and October 9, 2011, 21 WPV type I cases were confirmed in southern Xinjiang: 13 in Hotan, 6 in Kashgar, 1 in Bayinguole, and 1 in Akesu (Figure 1). Of the 21 WPV cases, 6 cases were less than 1 year of age, 4 were 1–4 years of age, and 11 cases occurred among adults (15–53 years old). The incidences of WPV (3.46/100,000) were highest among children <1 year of age.

### Study population

After the WPV outbreak was confirmed, a total of 2,611 participants were enrolled in 5 southern prefectures in Xinjiang Uygur Autonomous Region before SIAs were conducted: 400 in Akesu Prefecture, 510 in Bayinguole Prefecture, 930 in Hotan Prefecture, 359 in Kashgar Prefecture, and 412 in Kezilesukeer Prefecture (Table 1). Of the 2,611 participants, 198 were <1 years of age, 435 between 1 and 4 years, 596 between 5 and 14 years, 1059 between 15 and 39 years, and 323 were ≥40 years of age (Table 2). There was significant difference in age distribution among different prefectures (*P* = 0.02), with the highest median age (19.0±26.0) in Bayinguole Prefecture and lowest median age (14.5±24.0) in Akesu Prefecture. A total of 1214 (46.5%) were male, and there was statistically significant difference in gender proportions among prefectures (*P*<0.01).

**Table 1:** Polio antibody seropositivity of single serotype and combined serotypes, and GMTs of P1, P2 and P3 by prefecture in southern Xinjiang Uygur Autonomous Tegion before SIAs, 2011.

Polio serotypes	Akesu (N = 400)	Bayinguole (N = 510)	Hotan (N = 930)	Kashgar (N = 359)	Kezilesukeer (N = 412)	*P* value	Total (N = 2611)
Polio antibody seropositivity N (%)							
P1	353 (88.3)	417 (81.8)	816 (87.7)	319 (88.9)	348 (84.5)	<0.01	2253 (86.3)
P2	356 (89.0)	427 (83.7)	824 (88.6)	322 (89.7)	354 (85.9)	0.03	2283 (87.4)
P3	323 (80.8)	351 (68.8)	729 (78.4)	284 (79.1)	302 (73.3)	<0.01	1989 (76.2)
P1 & P2	327 (81.8)	370 (72.5)	755 (81.2)	297 (82.7)	315 (76.5)	<0.01	2064 (79.1)
P1 & P3	302 (75.5)	319 (62.5)	678 (72.9)	266 (74.1)	274 (66.5)	<0.01	1839 (70.4)
P2 & P3	301 (75.3)	318 (62.4)	688 (74.0)	268 (74.7)	281 (68.2)	<0.01	1856 (71.1)
P1& P2 & P3	286 (71.5)	296 (58.0)	649 (69.8)	254 (70.8)	259 (62.9)	<0.01	1744 (66.8)
P1 or P2 or P3	388 (97.0)	484 (94.9)	897 (96.5)	348 (96.9)	393 (95.4)	0.36	2510 (96.1)
GMTs of P1, P2 and P3							
P1	48.7	34.0	43.2	36.8	29.4	<0.01	38.6
P2	37.1	29.4	36.1	31.8	27.2	<0.01	32.7
P3	22.9	12.9	18.5	19.1	15.9	<0.01	17.5

Note: P1: poliovirus serotype 1; P2: poliovirus serotype 2; P3: poliovirus serotype 3.

SIAs: supplementary immunization activities. GMTs: geometric mean titres.

**Table 2:** Antibody seropositivity and GMTs of P1, P2 and P3 by demographic characteristics in southern Xinjiang Uygur Autonomous Region before SIAs, 2011.

			Total population N (%)	P1				P2				P3			
				Seropositive N (%)	*P* value	GMTs§	*P* value	Seropositive N (%)	*P* value	GMTs§	*P* value	Seropositive N (%)	*P* value	GMTs§	*P* value
Akesu Prefecture	Sex	Male	196 (49.0)	172 (87.8)	0.76	48.0	0.87	173 (88.3)	0.65	38.5	0.65	162 (82.7)	0.34	24.3	0.48
		Female	204 (51.0)	181 (88.7)		49.4		183 (89.7)		35.9		161 (78.9)		21.6	
	Age group(Yrs)	<1	34 (8.5)	24 (70.6)	<.01	25.1^a^	<.01	23 (67.6)	<.01	29.5^a^	<.01	20 (58.8)	<.01	18.8^a^	<.01
		1–4	87 (21.8)	83 (95.4)		187.3^b^		85 (97.7)		107.4^b^		83 (95.4)		77.5^b^	
		5–14	79 (19.8)	77 (97.5)		89.1^c^		79 (100.0)		50.5^c^		68 (86.1)		25.5^c^	
		15–39	159 (39.8)	131 (82.4)		23.2^a^		132 (83.0)		20.8^a^		114 (71.7)		12.3^a^	
		≥40	41 (10.3)	38 (92.7)		27.0^a^		37 (90.2)		24.9^a^		38 (92.7)		18.7^a^	
	Districts	Akesu City	150 (37.5)	128 (85.3)	0.25	36.0	0.05	128 (85.3)	0.27	32.5	0.07	116 (77.3)	0.33	19.5	0.18
		Awati County	50 (12.5)	44 (88.0)		66.7		46 (92.0)		58.1		39 (78.0)		22.9	
		Kuche County	150 (37.5)	133 (88.7)		54.4		135 (90.0)		34.1		124 (82.7)		23.4	
		Wensu County	50 (12.5)	48 (96.0)		63.1		47 (94.0)		45.9		44 (88.0)		34.9	
Bayinguole Prefecture	Sex	Male	230 (45.1)	186 (80.9)	0.64	37.5	0.27	192 (83.5)	0.89	29.5	0.96	164 (71.3)	0.27	14.3	0.20
		Female	280 (54.9)	231 (82.5)		31.4		235 (83.9)		29.3		187 (66.8)		11.9	
	Age group(Yrs)	<1	46 (9.0)	29 (63.0)	<.01	19.8^a^	<.01	34 (73.9)	<.01	32.0^a^	<.01	23 (50.0)	<.01	15.8^a^	<.01
		1–4	93 (18.2)	81 (87.1)		104.7^b^		79 (84.9)		74.3^b^		79 (84.9)		37.7^b^	
		5–14	76 (14.9)	71 (93.4)		74.0^b^		74 (97.4)		44.8^a^		65 (85.5)		14.9^a^	
		15–39	219 (42.9)	170 (77.6)		19.5^a^		184 (84.0)		21.6^c^		141 (64.4)		9.1^c^	
		≥40	76 (14.9)	66 (86.8)		27.2^a^		56 (73.7)		14.2^c^		43 (56.6)		7.2^c^	
	Districts	Bazhou City	98 (19.2)	83 (84.7)	<.01	31.3^a,b^	<.01	85 (86.7)	<.01	28.0^c,d^	<.01	62 (63.3)	<.01	9.3^b^	<.01
		Hejing County	20 (3.9)	17 (85.0)		28.8^a,b^		16 (80.0)		14.4^d^		14 (70.0)		14.4^a,b^	
		Kuerle County	100 (19.6)	88 (88.0)		43.1^a,b^		88 (88.0)		40.5^a,c^		78 (78.0)		15.2^a,b^	
		Luntai County	47 (9.2)	41 (87.2)		30.2^a,b^		44 (93.6)		39.9^a,c^		41 (87.2)		26.4^a^	
		Qiemo County	45 (8.8)	18 (40.0)		16.2^b^		15 (33.3)		7.1^b^		16 (35.6)		8.1^b^	
		Ruoqiang County	100 (19.6)	93 (93.0)		67.2^a^		94 (94.0)		58.5^a^		77 (77.0)		17.1^a,b^	
		Yanqi County	100 (19.6)	77 (77.0)		22.5^b^		85 (85.0)		21.3^c,d^		63 (63.0)		9.7^b^	
Hotan Prefecture	Sex	Male	447 (48.1)	394 (88.1)	0.72	41.6	0.57	401 (89.7)	0.31	37.8	0.41	348 (77.9)	0.70	18.5	0.98
		Female	483 (51.9)	422 (87.4)		44.7		423 (87.6)		34.5		381 (78.9)		18.5	
	Age group(Yrs)	<1	39 (4.2)	24 (61.5)	<.01	15.4^a^	<.01	27 (69.2)	<.01	15.7^a^	<.01	22 (56.4)	<.01	10.8^a^	<.01
		1–4	114 (12.3)	106 (93.0)		159.3^b^		104 (91.2)		93.3^b^		104 (91.2)		49.3^b^	
		5–14	294 (31.6)	275 (93.5)		76.6^c^		276 (93.9)		55.3^c^		237 (80.6)		22.8^c^	
		15–39	385 (41.4)	322 (83.6)		23.6^a^		333 (86.5)		23.8^a^		288 (74.8)		12.9^a^	
		≥40	98 (10.5)	89 (90.8)		27.6^a^		84 (85.7)		23.6^a^		78 (79.6)		16.2^a,c^	
	Districts	Cele County	316 (34.0)	249 (78.8)	<.01	20.7^d^	<.01	248 (78.5)	<.01	17.7^d^	<.01	208 (65.8)	<.01	10.9^c^	<.01
		Hotan City	76 (8.2)	73 (96.1)		290.9^a^		73 (96.1)		100.1^b^		67 (88.2)		38.8^b^	
		Luopu County	61 (6.6)	58 (95.1)		211.0^a^		57 (93.4)		168.1^a^		58 (95.1)		95.3^a^	
		Minfeng County	221 (23.8)	198 (89.6)		22.7^d^		205 (92.8)		31.7^c^		175 (79.2)		14.7^c^	
		Moyu County	88 (9.5)	84 (95.5)		109.3^b^		83 (94.3)		63.5^b^		78 (88.6)		31.0^b^	
		Pishan County	107 (11.5)	98 (91.6)		39.4^c^		99 (92.5)		29.4^c^		90 (84.1)		14.7^c^	
		Yutian County	61 (6.6)	56 (91.8)		116.9^b^		59 (96.7)		88.0^b^		53 (86.9)		36.7^b^	
Kashgar Prefecture	Sex	Male	170 (47.4)	143 (84.1)	<.01	28.9	<.01	150 (88.2)	0.39	30.6	0.65	123 (72.4)	<.01	15.6	0.01
		Female	189 (52.6)	176 (93.1)		45.7		172 (91.0)		32.9		161 (85.2)		23.0	
	Age group(Yrs)	<1	40 (11.1)	30 (75.0)	0.02	14.2^a^	<.01	27 (67.5)	<.01	12.3^a^	<.01	15 (37.5)	<.01	7.0^a^	<.01
		1–4	61 (17.0)	55 (90.2)		77.6^b^		56 (91.8)		52.2^b^		52 (85.2)		28.6^b^	
		5–14	65 (18.1)	62 (95.4)		67.5^b^		63 (96.9)		59.3^b^		56 (86.2)		28.5^b^	
		15–39	133 (37.0)	116 (87.2)		24.0^a^		119 (89.5)		28.5^c^		105 (78.9)		16.0^b^	
		≥40	60 (16.7)	56 (93.3)		43.2^b^		57 (95.0)		23.4^c^		56 (93.3)		24.3^b^	
	Districts	Jiashi County	100 (27.9)	92 (92.0)	0.57	44.6^a^	0.04	90 (90.0)	0.76	37.0^a^	0.01	88 (88.0)	0.04	22.2	0.39
		Kashgar City	99 (27.6)	87 (87.9)		24.5^a^		91 (91.9)		20.4^b^		77 (77.8)		15.7	
		Shache County	100 (27.9)	86 (86.0)		40.5^a^		89 (89.0)		37.8^a^		77 (77.0)		20.7	
		Yecheng County	60 (16.7)	54 (90.0)		44.2^a^		52 (86.7)		38.4^a^		42 (70.0)		18.4	
Kezilesukeer Prefecture	Sex	Male	171 (41.5)	142 (83.0)	0.50	31.1	0.54	141 (82.5)	0.09	26.2	0.66	122 (71.3)	0.45	15.8	0.97
		Female	241 (58.5)	206 (85.5)		28.2		213 (88.4)		28.0		180 (74.7)		15.9	
	Age group(Yrs)	<1	39 (9.5)	24 (61.5)	<.01	23.2^a,b^	<.01	22 (56.4)	<.01	13.3^a^	<.01	23 (59.0)	<.01	16.3^a^	<.01
		1–4	80 (19.4)	70 (87.5)		74.2^c^		71 (88.8)		58.2^b^		66 (82.5)		33.7^b^	
		5–14	82 (19.9)	78 (95.1)		39.5^b^		76 (92.7)		32.0^c^		70 (85.4)		19.4^a^	
		15–39	163 (39.6)	133 (81.6)		17.8^a^		142 (87.1)		19.9^a,c^		105 (64.4)		10.2^a^	
		≥40	48 (11.7)	43 (89.6)		25.0^a,b^		43 (89.6)		30.2^c^		38 (79.2)		13.8^a^	
	Districts	Aheqi County	62 (15.0)	58 (93.5)	0.13	47.3^a^	0.02	62 (100.0)	<.01	47.9^a^	<.01	56 (90.3)	<.01	31.3^a^	<.01
		Aketao County	100 (24.3)	87 (87.0)		33.6^a,b^		90 (90.0)		29.4^a,b^		81 (81.0)		21.1^a^	
		Atushi County	100 (24.3)	79 (79.0)		23.1^b^		76 (76.0)		20.8^b^		57 (57.0)		10.7^b^	
		Kezilesukeer City	100 (24.3)	82 (82.0)		22.3^b^		83 (83.0)		23.1^b^		69 (69.0)		10.8^b^	
		Wuqia County	50 (12.1)	42 (84.0)		34.8^a,b^		43 (86.0)		27.5^a,b^		39 (78.0)		18.4^a,b^	

Note: P1: poliovirus serotype 1; P2: poliovirus serotype 2; P3: poliovirus serotype 3.

SIAs: supplementary immunization activities. GMTs: geometric mean titres.

§ SNK post hoc tests were performed to detect the inter-group difference if significant difference was present in ANOVA, with different letters indicating statistically significant difference.

### Polio antibodies seroprevalences and GMTs

Among the 2,611 participants enrolled, 2,253 (86.3%), 2,283 (87.4%), and 1,989 (76.2%) were seropositive to P1, P2 and P3 respectively at titers ≥1∶8, and GMTs of P1, P2 and P3 were 38.6, 32.7 and 17.5 respectively (Table 1). There were significant differences in antibody seropositivity and GMTs among P1, P2 and P3, with lower antibody seropositivity and GMTs observed for P3. With regard to seropositivity to more than one serotype, 2,064 (79.1%) were seropositive to a combination of P1 & P2 serotypes, while 1,839 (70.4%) and 1,856 (71.1%) were seropositive to a combination of P1 & P3, and P2 & P3 respectively at titers ≥1∶8. Overall, only 1744 (66.8%) participants were positive to all the three serotypes: 286 (71.5%) in Akesu Prefecture, 296 (58.0%) in Bayinguole Prefecture, 649 (69.8%) in Hotan Prefecture, 254 (70.8%) in Kashgar Prefecture, and 259 (62.9%) in Kezilesukeer Prefecture. The lowest seropositive rates were observed in Bayinguole Prefecture for P1, P2, P3 and any combination of three serotypes (including P1 & P2, P1 & P3, P2 & P3, P1 & P2 & P3), and was significant lower than seropositive rates observed in other 4 prefectures. And the lowest GMTs were also found in Bayinguole Prefecture for P1, P2, and P3.

**Figure 2:**
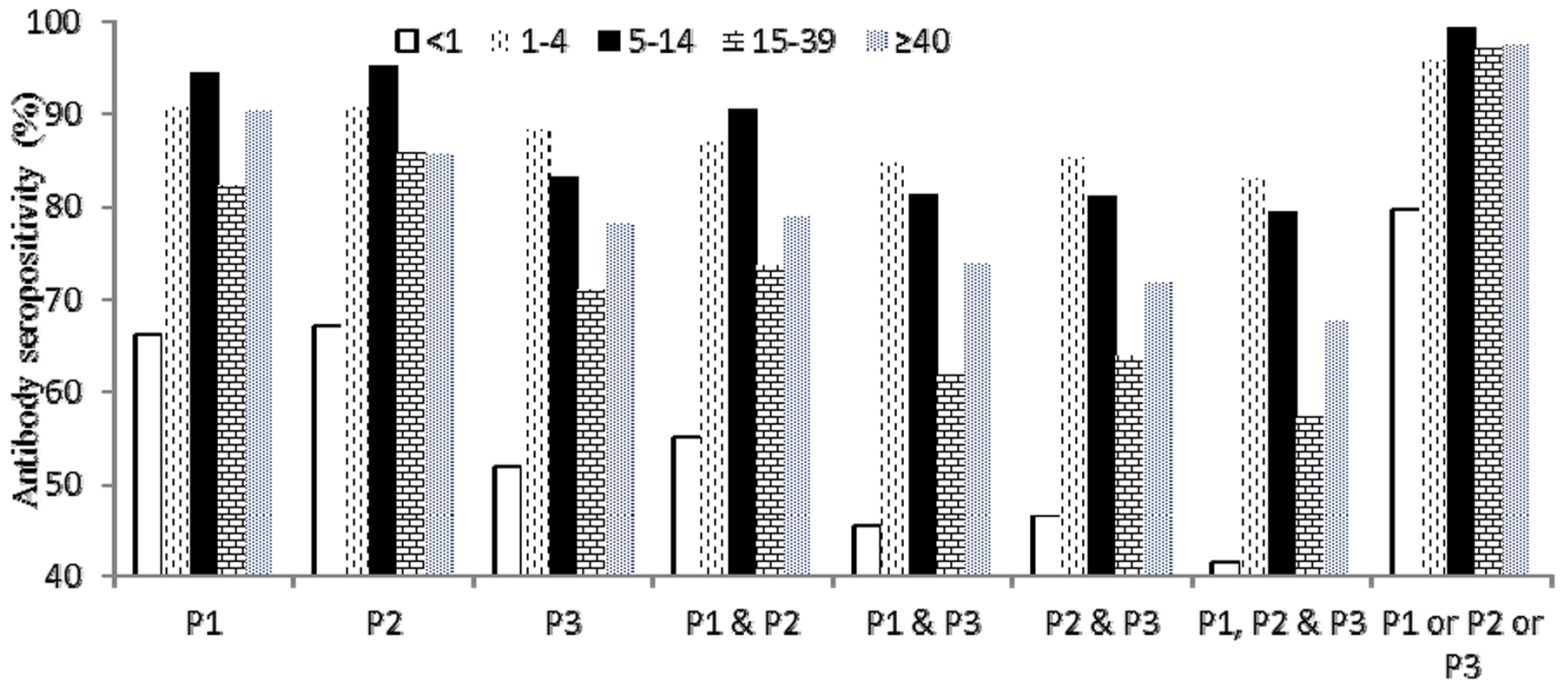
Antibody seropositivity of single serotype and combined serotypes by age group in southern Xinjiang Uygur Autonomous Region before SIAs, 2011. Note: P1: poliovirus serotype 1; P2: poliovirus serotype 2; P3: poliovirus serotype 3.

**Figure 3:**
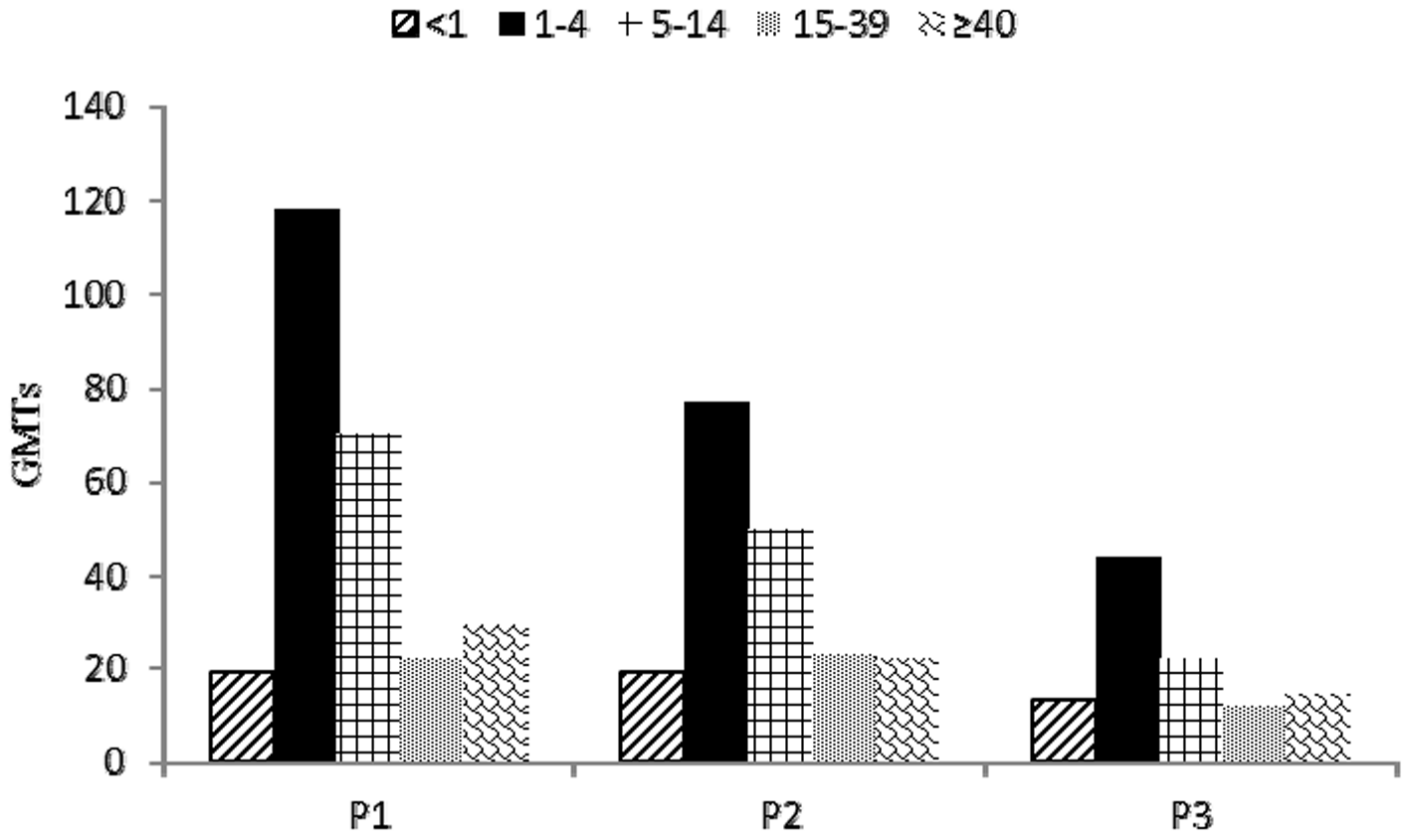
GMTs of P1, P2 and P3 by age group in southern Xinjiang Uygur Autonomous Region before SIAs, 2011. Note: P1: poliovirus serotype 1; P2: poliovirus serotype 2; P3: poliovirus serotype 3.

We observed a correlation between age groups and seropositivity. For P1 and P2, the antibody seroprevalence was highest in children aged 5–14 years and was >80% in the majority of age groups (Figure 2), except for children <1 year of age. For P3 we observed lower antibody seroprevalences in all age groups, and the highest P3 seropositivity was in children aged 1–4 years. For all 3 serotypes of poliovirus, the lowest antibody seroprevalence was lowest in children <1 year of age, and the secondary lowest antibody seroprevalence was in adults aged 15–39 years. With regard to antibodies to more than one serotype, a similar trend was found with lower seroprevalence in children <1 year of age and in adults aged 15–39 years. Seropositivity for P1 & P2 & P3 was less than 50% in children <1 year of age and less than 60% in adults aged 15–39 years. We also found lower GMTs of P1, P2, P3 in children <1 year of age and in adults aged 15–39 years (Figure 3).

The relationships between demographic characteristics and antibody GMTs by prefecture are shown in Table 2. There was no statistically significant difference of antibody seropositivity and GMTs between male and female participants for all 3 serotypes in almost all the prefectures, except for P1 and P3 in Kashgar Prefecture where higher antibody seropositivity and GMTs for P1 and P3 were observed among female participants. We did observe a correlation among age groups and seropositivity in each prefecture, again with the lower antibody seropositivity for all 3 serotypes poliovirus in children <1 year of age and in adults aged 15–39 years, except for P2 and P3 in Bayinguole Prefecture where antibody seropositivities of P2 and P3 in adults ≥ years old were lower than in adults aged 15–39 years old. Similar differences of GMTs among age groups were also found.

In Bayinguole Prefecture, there was statistically significant difference in antibody seropositivity and GMTs among districts, with lowest antibody seropositivity and GMTs in Qiemo County where unique WPV case was found in Bayinguole Prefecture. In Qiemo County, an extremely low antibody seropositivity was observed (40.0% for P1, 33.3% for P2 and 35.6% for P3). In Hotan Prefecture, there was statistically significant difference in antibody seropositivity and GMTs among districts, with lowest antibody seropositivity and GMTs in Cele County. In Kashgar Prefecture, for P1 and P2, there was no statistically significant difference in antibody seropositivity among districts, but P3 antibody seropositivity was significant higher in Jiashi County than in other three counties; there was statistically significant difference of GMTs for P1 and P2, but not for P3. In Kezilesukeer Prefecture, there was statistically significant difference in antibody seropositivity and GMTs among districts, with lower antibody seropositivity and GMTs in Atushi County. There was no statistically significant difference of antibody seropositivity and GMTs among different counties in Akesu Prefecture.

## Discussion

This survey was conducted immediately after the confirmation of WPV outbreak but before SIAs conducted in southern Xinjiang Uygur Autonomous Region [4]. Data from this study were useful in identifying potentially susceptible groups and guiding preparedness and response plan. A comparatively low seroprevalence of polio antibody was found in our study: 66.8% of participants were seropositive to all the three serotypes of poliovirus, and 86.3%, 87.4%, and 76.2% of participants were seropositive to P1, P2 and P3 respectively. A correlation between age groups and seropositivity in all or by prefecture level was observed in the study: both highest seropositive rates and GMTs were among children aged 5–14 years, while lower seroprevalence was found in children <1 year of age and in adults aged 15–39 years.

Only 66.8% participants were seropositive to all the three serotypes of poliovirus in southern Xinjiang Uygur Autonomous Region, which implied low population immunity and high risk of WPV transmission if WPV was imported. Both high antibody seropositivity and GMTs for all 3 serotypes of poliovirus were found among children aged 5–14 years, in which only 1 clinical compatible polio case was reported (unpublished data). Lowest seroprevalence was found in children <1 year of age and followed by adults aged 15–39 years. Accordingly, the highest incidence of WPV and clinical compatible polio cases were reported in children <1 year of age, while most of WPV cases and clinical compatible polio cases were reported in adults aged 15–39 years: 47.6% (10/21) WPV cases and 60.9% (14/23) clinical compatible polio cases [16]. Lowest antibody seropositivity (40.0% for P1, 33.3% for P2 and 35.6% for P3) and GMTs were found in Qiemo County where a unique WPV case was found in Bayinguole Prefecture.

It was easy to understand that children <1 year of age had lowest seroprevalence as they may not yet be scheduled for vaccination or had delays in vaccination. However, it is unclear why adults aged 15–39 years also had lower antibody seroprevalence and accounted most of WPV cases and clinically compatible polio cases, especially compared with the older population. Similarly, other countries with no recent WPV transmission have faced similar outbreaks characterized by a large proportion of cases in older age groups, such as Albania in 1996 [18], Cape Verde in 2000 [19]–[21], Namibia in 2006 [22], [23], and the Republic of the Congo in 2010 [24]. In Namibia, 14/19 (74%) confirmed cases were adults aged 15–29 years in 2006 [22], [23]. In addition to vaccination, a further method of protection is past exposure to WPV strains. To our knowledge, transmission of WPV have greatly reduced since the early 1980s in Xinjiang Uygur Autonomous Region, decreasing opportunities for unvaccinated populations to acquire natural immunity, moreover, the actual coverage of routine immunization was not high enough, these factors possibly contributing to lower antibody seroprevalence in adults aged 15–39 years. Low vaccination coverage and no previous exposure to wild virus likely led to an accumulation of susceptible persons.

As shown in other studies [12], [24], [25], serological surveys can be used to identify susceptible population with immunity gaps to polio, and subsequently to guide vaccination campaigns targeting high-risk population. Based on our serosurvey results, the targeted populations for SIAs were expanded to <40 years in Southern Xinjiang, thereafter, we conducted SIAs targeting adults aged 15–39 years old in Southern Xinjiang (Hotan on Sept. 13–17, and the other four southern prefectures on Sept. 22–26) immediately after the first round of SIA targeting children <15 years old which were conducted between Sept. 8–12, 2011. The fact that the last WPV cases were paralyzed on Oct. 9, 2011 (just at the beginning of second rounds of SIAs which was initiated on Oct. 8, 2011), suggests that transmission of WPV might have been interrupted by the first round of SIAs due to the high quality of the acute flaccid paralysis surveillance system. The success of quickly interrupting WPV transmission reinforces our decision on conducting SIAs for adults in Southern Xinjiang.

Lower antibody seroprevalence and GMTs were observed for P3 in each age group. The decrease of seroprevalence for P3 was also showed similarly in other serological studies [26]–[36], particularly in teenagers and young adults [29], [34]. It may be explained by a lower potency of P3 antigens in trivalent OPV, with suboptimal levels of protection for P3 following OPV immunization [37]. Moreover, humoral immunity to P3 declined significantly over time [38], and P3 antibody titers may eventually decrease below the detection level [39]. The waning effect of polio antibody may also contribute the low antibody seropositivity in adults aged 15–39 years.

Our study has several limitations. The study was based on convenience sampling, whether the seroprevalence of antibody against poliovirus among enrolled participants who hospitalized at county-level or above was representative of the community is uncertain. Participants were enrolled in areas affected by WPV to determine the population immunity in these areas. Therefore, the sampled population in our study is not necessarily representative of the overall population in southern Xinjiang, and limits the generalizability of our findings.

## Conclusions

In many districts of the world without WPV transmission, immunization services remain weak. Low vaccination coverage and no previous exposure to WPV likely led to an accumulation of susceptible participants. Once introduced, WPV can spread quickly through this highly susceptible young adult population [24], [25]. Especially given the waning immunity, consideration should be given for inclusion of a booster dose of OPV for teenagers [40], and SIAs should be expanded to adults if possible when WPV importation into countries without WPV transmission for many years occurs. Consideration should be given during polio risk assessment planning and outbreak response to potential susceptibility to polio in older children and adults. Serological surveys can be used to identify susceptible population with immunity gaps to polio, and subsequently to guide vaccination campaigns targeting high-risk population.
